# Interfaces between language and cognition

**DOI:** 10.3389/fpsyg.2013.00258

**Published:** 2013-05-06

**Authors:** Andriy Myachykov, Christoph Scheepers, Yury Y. Shtyrov

**Affiliations:** ^1^Department of Psychology, Northumbria UniversityNewcastle upon Tyne, UK; ^2^Institute of Neuroscience and Psychology, University of GlasgowGlasgow, UK; ^3^MRC Cognition and Brain Sciences Unit, Cambridge UniversityCambridge, UK

One of the most intriguing and challenging questions in the interdisciplinary study of mental processes and underlying brain mechanisms is how language is related to thought. The question is by no means new. Scholars have attempted to unravel the relationship between language and thought since the early days of Western philosophy. Recent theories range from strictly modular accounts of linguistic processing to fully integrated theories, according to which linguistic processes strongly interact with more general cognitive mechanisms such as attention, memory, and action control. Unfortunately, theoretical exchange between proponents of these different views is often lacking. In part, this is due to the interdisciplinary nature of the question itself. As a result, researchers representing various disciplines often fail to engage in an exchange of theoretical views, research ideas, and methodological expertise. The present Frontiers' Special Topic provides a platform for such dialogue. It features contributions discussing the latest advances and challenges in the frontline research on language and cognition and attempts to provide a joint discussion forum for a wide range of researchers from the domains of cognitive psychology, neuroscience, and psycholinguistics, among others. These researchers follow different theoretical approaches and use different experimental methodologies. What unites them is their goal to understand the mechanisms underlying the interplay between linguistic and general cognitive processes.

General cognitive mechanisms in linguistic communication do not only include retrieval and processing of linguistic information; they also rely upon constant updating and organizing of this linguistic information in relation with other, more general representations. Some existing theoretical models assume a tight interactive coupling between domain-general and domain-specific sources of information in the cognitive organization of the linguistic faculty. Domain-specific constraints may include, for example, grammatical as well as lexical and pragmatic knowledge. Domain-general constraints comprise processing limitations imposed by the cognitive mechanisms of memory, attention, learning, and social interaction. However, much of the existing research tends to focus on one or the other of the aforementioned areas, while integrative accounts are still rather sparse at present. The aim of this Special Topic of Frontiers in Cognition is therefore to bring together researchers who, within their respective research fields and by using different methodologies, represent integrative approaches to the study of language. Our Research Topic presents a collection of seventeen excellent articles that include original research, commentaries, opinions, and reviews.

A number of papers in this Topic discuss neurophysiological and behavioral evidence about the interface between language, perception, and attention. Research discussed by Roelofs and Piai (2011) suggests that word planning does not always require full executive attention while specific attention deficits may contribute to impaired language performance. The results discussed by Roelofs and Piai (2011) demonstrate how gaze shifts can be linked to the process of phonological encoding with specific focus on word production automaticity. The article by Myachykov et al. (2012) presents evidence about the special role attention plays in determining the assignment of grammatical roles and the associated syntactic choice in visually situated sentence production. Papers by Huettig et al. (2012), Knoeferle et al. (2011), and Kaiser (2012) provide complementary evidence about the involvement of the language-cognition interface during sentence comprehension in visually situated contexts. The contribution by Shtyrov (2011) reports novel findings about rapid pre-attentive mapping of novel word forms, as evidenced by changes in the dynamics of brain responses within very short exposures. Finally, Hussey and Novick (2012) report intriguing evidence about the benefits of executive control training for grammatical processing in ambiguous contexts.

The question of coordination between interlocutors during dialogue is raised in two articles. Gambi and Pickering (2011) used a novel interactive methodology in order to demonstrate that interlocutors constantly coordinate their sentences to represent their partner's knowledge. They then use these representations to build unfolding predictions, which they take into account when planning self-generated utterances. Similarly, Dale and colleagues (2011) use eye-movement synchronization between interlocutors as evidence for rapid approximation of actions in dialogue and the emergence of a single coordinated interactive system.

Three papers in our Topic discuss embodied and grounded aspects of language processing. Lupyan (2012) addresses the question of the language-cognition interplay from the point of view of how language affects cognition and perception. In particular, Lupyan (2012) reviews evidence showing that performance on tasks that have been presumed to be non-verbal is rapidly modulated by language. Klemfuss et al. (2012) discuss effects of language on perception by critically reviewing evidence suggesting top-down influences of linguistic representations on visual feature detection. Their own research suggests that visual search is disrupted by the automatic activation of irrelevant linguistic representations. Another important aspect of the grounded view of language is the role played by perception and action systems in the organization of abstract knowledge. Scorolli et al. (2011) discuss the crucial role played by embodied theories of cognition in linguistic experience for abstract words.

A number of papers in this Special Topic discuss architectural properties of the language-cognition interface. For example, Menenti et al. (2012) investigated how brain areas adapt to repetition of various sentence properties, thereby unraveling the neuronal infrastructure for the specific components of semantic encoding. Mashal and colleagues (2012) present a novel cortical network model for observation and imitation of speech. Their results show that the network models for observation and imitation comprise the same essential structure but differ in important features that reflect distinct connectivity patterns. Andric and Small (2012) contribute to the debate by discussing how the brain processes language and co-occurring gestures. Finally, Naylor et al. (2012) focus on cognitive and electrophysiological correlates of the bilingual Stroop effect by analysing corresponding ERP components in bilingual speakers. Their research shows, among other things that color words from both languages created response conflict and that the between-within language Stroop effect reflects complex brain activity with contributions from language both and color at different task points.

## References

[B1] Andric M. Small S. L. (2012). Gesture's neural language. Front. Psychol. 3:99 10.3389/fpsyg.2012.00099 22485103 PMC3317265

[B2] Dale R. Kirkham N. Z. Richardson D. C. (2011). The dynamics of reference and shared visual attention. Front. Psychol. 2:355 10.3389/fpsyg.2011.00355 22164151 PMC3230789

[B3] Gambi C. Pickering M. J. (2011). A cognitive architecture for the coordination of utterances. Front. Psychol. 2:275 10.3389/fpsyg.2011.00275 22065961 PMC3206582

[B4] Huettig F. Mishra R. K. Olivers C. N. L. (2012). Mechanisms and representations of language-mediated visual attention. Front. Psychol. 2:394 10.3389/fpsyg.2011.00394 22291672 PMC3253411

[B5] Hussey E. K. Novick J. M. (2012). The benefits of executive control training and the implications for language processing. Front. Psychol. 3:158 10.3389/fpsyg.2012.00158 22661962 PMC3356880

[B6] Kaiser E. (2012). Taking action: a cross-modal investigation of discourse-level representations. Front. Psychol. 3:156 10.3389/fpsyg.2012.00156 22701440 PMC3372065

[B7] Klemfuss N. Prinzmetal W. Ivry R. B. (2012). How does language change perception: a cautionary note. Front. Psychol. 3:78 10.3389/fpsyg.2012.00078 22454625 PMC3308142

[B8] Knoeferle P. Carminati M. N. Abashidze D. Essig K. (2011). Preferential inspection of recent real-world events over future events: evidence from eye tracking during spoken sentence comprehension. Front. Psychol. 2:376 10.3389/fpsyg.2011.00376 22207858 PMC3245670

[B9] Lupyan G. (2012). Linguistically modulated perception and cognition: the label-feedback hypothesis. Front. Psychol. 3:54 10.3389/fpsyg.2012.00054 22408629 PMC3297074

[B10] Mashal N. Solodkin A. Dick A. S. Chen E. E. Small S. L. (2012). A network model of observation and imitation of speech. Front. Psychol. 3:84 10.3389/fpsyg.2012.00084 22470360 PMC3312271

[B11] Menenti L. Petersson K. M. Hagoort P. (2012). From reference to sense: how the brain encodes meaning for speaking. Front. Psychol. 2:384 10.3389/fpsyg.2011.00384 22279438 PMC3260530

[B12] Meyer A. S. Wheeldon L. van der Meulen F. Konopka A. (2012). Effects of speech rate and practice on the allocation of visual attention in multiple object naming. Front. Psychol. 3:39 10.3389/fpsyg.2012.00039 22363310 PMC3282304

[B13] Myachykov A. Thompson D. Garrod S. Scheepers C. (2012). Referential and visual cues to structural choice in visually situated sentence production. Front. Psychol. 2:396 10.3389/fpsyg.2011.00396 22279439 PMC3260531

[B14] Naylor L. J. Stanley E. M. Wicha N. Y. Y. (2012). Cognitive and electrophysiological correlates of the bilingual stroop effect. Front. Psychol. 3:81 10.3389/fpsyg.2012.00081 22485099 PMC3317261

[B15] Roelofs A. Piai V. (2011). Attention demands of spoken word planning: a review. Front. Psychol. 2:307 10.3389/fpsyg.2011.00307 22069393 PMC3209602

[B16] Scorolli C. Binkofski F. Buccino G. Nicoletti R. Riggio L. Borghi A. M. (2011). Abstract and concrete sentences, embodiment, and languages. Front. Psychol. 2:227 10.3389/fpsyg.2011.00227 21954387 PMC3173827

[B17] Shtyrov Y. (2011). Fast mapping of novel word forms traced neurophysiologically. Front. Psychol. 2:340 10.3389/fpsyg.2011.00340 22125543 PMC3221415

